# Prof. Bigelow’s Intoductory Lecture—Clinical Lectures

**Published:** 1850-05

**Authors:** 


PROF. BIGELOW'S INTRODUCTORY LECTURE.—CLINICAL LECTURES.

We are indebted to Henry J. Bigelow, M. D., Professor of Surgery in Harvard University, for a copy of his excellent introductory lecture. We are especially pleased with the forcible and convincing manner in which Dr. Bigelow urges the importance of clinical instruction. On this subject there should be no difference of opinion among medical men; all should unite in an earnest endeavor to advance the interests of clinical instruction. One good clinical lecture may be worth scores of didactic performances, no matter how able the latter may be. We would not disparage the value of didactic instruction; it is valuable, nay even essential in its place, but its importance is greatly lessened without it is connected with clinical teaching. And we confidently indulge the hope that the time will come, when the absurdity of attempting to teach medicine without clinics will be universally scouted.

On the subject of clinical instruction, Dr. Bigelow thus discourses:

"It has come to be questioned how far clinical instruction is essential to a course of medical teaching. Local interests or local exigencies have led to a discussion of the value of this method of imparting knowledge, and as seriously as if there were some doubt about it. Surely those who hesitate, do not consider the difference between words and things; between the aspect of a man himself and such a detailed description of him as the police might give; between visible and tangible disease, and a written history of it. No doubt an original fact and its description both gain access to the understanding; but there is a difference in the quality of the knowledge thus obtained. To value a possession, the mind must first have felt the want of it. Curiosity must first stimulate both its perception and its ability to retain. The mind asks a question, and is then polarized for the reception of a direct answer; and it is balked and wearied by an irrelevant reply. Now every protracted description, especially a lecture, is of the nature of a series of replies to which no question has been asked. A whole audience cannot ask or be answered at one time, and the alternative is to distribute information in bulk, that each may select something which will approximate his purpose.

"On the other hand, exhibit a case of actual disease, and every observer will put and answer in his own mind, and with the rapidity of thought, an endless variety of queries upon points in which, perhaps, he alone is deficient, and for the reception of which his mind alone is stimulated.

"Another point is more important. Sensible qualities must be described by reference to acknowledged standards; and we can thus measure heat, and space, and weight; but not shades of color, nor the attributes soft and hard, nor the varying outline of a curve. In the same way a personal examination will yield the qualities of an odor, a pulse, a tumor, an expression of the features, which pages of tedious description might fail to do. And the mind which painfully contemplates an abstraction, will seldom fail, at such a moment, to arrest some tangible association by which the abstract quality is permanently fixed.

"Clinical study is bed study. Here the student closes and grapples with the malady of whose Protean forms he has as yet only read. Here he learns at once the language of disease and the language of suffering humanity; and while his scientific sense is educated, his kindlier feelings are also developed. He learns to listen patiently, to sympathize; he learns to re-establish a facility in the manifestation of that stratum of kindly feeling which is generally upon the snrface in early youth, but which sometimes, in the process of education, gets imbedded beneath a show of indifference and insensibility.

"The dialect of disease is an especial object of clinical study. Is a fever settled? Is a cough seated on the lungs? Is there water on the brain? Such questions are as significant as if conveyed in the language of recondite science. On the other hand, there are propositions less intimately according with modern views. What is the cause of this? asks one. Is this a scrofula humor? Is it in my constitution? These, or even the vexed question of biliousness, may well perplex the votary of rigid science. Such queries suppose the physician to possess a truly intimate knowledge of the human frame. In the words of Sir Thomas Browne, two hundred years ago, 'They foolishly conceive that we visibly behold therein the anatomy of every particle, and can thereby indigitate their diseases; and running into any demands, expect from us a sudden resolution in things whereon the Devil of Delphos would demur; and we know hath taken respite of some days to answer easier questions."

In our judgment, these remarks present the subject in a proper point of view, and they merit the attention of all who have any interest in the subject of medical education. There are two important features of clinical teaching, on which every student should inform himself: there must be clinics to afford illustrations to the teacher, and there must be a teacher capable of making proper use of the cases. One of these features is as important as the other; and unless both are combined in one school, the whole attempt is a failure. The possession of multiplied and various specimens of diseased actions is of no great value without a teacher capable of making them speak in the instruction of students, nor is even a renowned teacher of much value without the clinical means. With these combined values, and a door thrown wide open to students who wish to enjoy the benefit of hospital instruction, a medical school should command confidence and public support.

In our former number we referred to Prof, Bartlett's clinical course especially, because our remarks were founded upon predictions made in the fall, and because he had been untried, in the West, in that department. We owe to Dr. David W. Yandell the expression of our thanks for the efficient and zealous aid he gave towards perfecting the course of clinical medicine, in the Marine Hospital, the past winter. He is entitled to the thanks of all who feel an interest in the progress of true science; that progress is based essentially on clinical instruction, and Dr. Yandlle's fine abilities in diagnosis, and in describing diseased movements have been conspicuously displayed during the recent course of medical lectures in this city.

An attempt is sometimes made, by those who have an itching palm for medical professorships, and very, very limited means for commanding the confidence of those desirous of being taught, to decry the common methods of clinical instruction, and the principle of *suppressio veri* is called into requisition. The representation is made that the only approach to clinical instruction is such as is obtained by hearing the lecturer describe the disease of the patient before the class. This is a manifest error. One of the chief values of hospital facilities is found in the means the students enjoy of seeing diseased actions in the hospital, and each one may make himself acquainted with the condition of every patient in the public wards. This, and the lectures of able clinical teachers constitute a fund of instruction that no falsehood can impair.

